# The complete mitochondrial genome of the hybrid of *Takifugu obscurus* (♀) × *Takifugu rubripes* (♂)

**DOI:** 10.1080/23802359.2019.1669082

**Published:** 2019-09-24

**Authors:** Dongyu Dou, Xiuli Wang, Haoyong Zhu, Yulong Bao, Yaohui Wang, Jun Cui, Xuesong Meng, Yongxiang Zhu, Xuemei Qiu

**Affiliations:** a College of Fisheries and Life Science, Dalian Ocean University, Dalian, 116023, Liaoning, China;; b Jiangsu Zhongyang Group Company Limited, Haian, Jiangsu, China;; c Dalian Tianzheng Industrial Co. Ltd, Dalian, Liaoning, China

**Keywords:** Mitochondrial genome, *Takifugu obscurus*, *Takifugu rubripes*, hybrid

## Abstract

The complete mitochondrial genome of the hybrid of *Takifugu obscurus* (♀) × *Takifugu rubripes* (♂) was sequenced and characterized in this study. The hybrid mitochondrial DNA, with a total length of 16,443 bp, contain 37 genes, including 13 protein-coding genes, 22 tRNAs genes, and 2 rRNA gene. There are two noncoding regions: the origin of light-strand replication (OL) and control region (D-loop). The complete mitochondrial genome of the hybrid of *T. obscurus* (♀) × *T. rubripes* (♂) provides data for studies on genetic diversity and species identification among *Takifugu*.

Both *Takifugu obscurus* and *Takifugu rubripes* belong to the family Tetrodontidae, mainly distribute in the Yellow Sea and Bohai Sea in China. *T. obscurus* and *T. rubripes* are the most important commercial *Takifugu* fishes in China because of their high nutritional value and deliciousness. The hybrid of *T. obscurus* (♀) × *T. rubripes* (♂) is obtained by artificial hybridization experiment, which has great potential economic value. Since mitochondrion plays an important role in the study of biological evolution, genetic diversity and species identification, it is necessary to conduct scientific research on mitochondrion of the hybrid (Jiang et al.^2016^; Li et al.^2016^).

The male *T. rubripes* originates from Dalian Tianzheng Industrial Co. Ltd, Dalian, China (121.34°E, 38.98°N). The female *T. obscurus* comes from Jiangsu Zhongyang Group Company Limited, Haian, China (120.91°E, 32.62°N). The hybrid experiments were conducted in Jiangsu Zhongyang Group Company Limited in April, 2018. The hybrid individuals of *T. obscurus* (♀) *× T. rubripes* (♂) were collected in August, 2018. The specimens (DLOU-MGL-20180801∼DLOU-MGL-20180803) were deposited in the Molecular Genetics Laboratory of Dalian Ocean University, Dalian, China. Nine pairs of primers were designed to clone the hybrid mitochondrial DNA according to the mitochondrial genome sequence of *T. rubripes* by molecular biotechnology. The complete mitochondrial DNA of the hybrid was sequenced and analyzed. The obtained mitochondrial genome sequence of the hybrid has been deposited to the GenBank database. The accession number is MK975474.

The hybrid mitochondrion, with a total length of 16443 bp, contain 37 genes, including 13 protein-coding genes, 22 tRNAs genes, one small subunit of the rRNA gene, and one large subunit of rRNA gene. There are 2 noncoding regions: origin of light-strand replication (OL) and control region (D-loop). All the genes conform to the typical mitochondrial gene arrangement in vertebrates (Prosdocimi et al. 2012; Zhang et al.^2016^). The hybrid mitochondrial genome composition is 30.02% for A, 25.74% for T, 29.13% for C, and 15.11% for G. The content of A + T (55.76%) is higher than the content of G + C (44.24%). Most of the genes are encoded on heavy chains (H-chains), except for 8 tRNAs (*Gln*, *Ala*, *Asn*, *Cys*, *Tyr*, *Ser*, *Glu*, and *Pro*) genes and 1 protein-coding gene (*ND6*). All protein initiation codons are ATG, except for COX1 starting with GTG. The phylogenetic relationship of the hybrid of *T. obscurus* (♀) × *T. rubripes* (♂) (MK975474) and other 16 tetrodontidae fish were analyzed by MEGA 7.0 software, using neighbor-joining method (Figure 1). Almost all tetrodontidae fish whose mitochondrial genome has been submitted in Genbank database were included. The result shows that the hybrid of *T. obscurus* (♀) × *T. rubripes* (♂) is more closely related to *T. obscurus* (female parent) than the other species. This is the first report on mitochondrial genome of the hybrid of *T. obscurus* (♀) × *T. rubripes* (♂), which provides a data for studies on genetic diversity and species identification among *Takifugu*.

**Figure 1.**
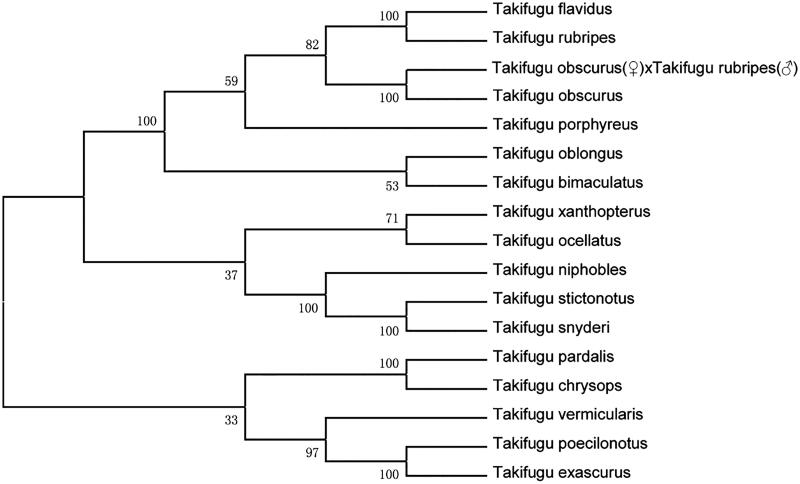
The evolutionary tree based on mitochondrial genome nucleotide sequences of the hybrid of *Takifugu obscurus* (♀) × *Takifugu rubripes* (♂) (MK975474) and other 16 tetrodontidae fish were constructed by MEGA 7.0 software, using neighbor-joining method. Genetic distances are listed above the branches. GenBank accession numbers of the sequences were used for the tree as follows: *Takifugu flavidus* (NC_024199.1); *Takifugu rubripes* (NC_004299.1); *Takifugu obscurus* (NC_011626.1); *Takifugu porphyreus* (NC_011628.1); *Takifugu oblongus* (NC_011634.1); *Takifugu bimaculatus* (KP973944.1); *Takifugu xanthopterus* (NC_011632.1); *Takifugu ocellatus* (NC_011635.1); *Takifugu niphobles* (NC_011625.1); *Takifugu stictonotus* (NC_011629.1); *Takifugu snyderi* (NC_011630.1); *Takifugu pardalis* (NC_011627.1); *Takifugu chrysops* (NC_011624.1); *Takifugu vermicularis* (NC_011631.1); *Takifugu poecilonotus* (NC_011621.1); *Takifugu exascurus* (AP009540).
